# “Rational Vaccine Design” for HIV Should Take into Account the Adaptive Potential of Polyreactive Antibodies

**DOI:** 10.1371/journal.ppat.1002095

**Published:** 2011-06-16

**Authors:** Jordan D. Dimitrov, Michel D. Kazatchkine, Srinivas V. Kaveri, Sebastien Lacroix-Desmazes

**Affiliations:** 1 INSERM U872, Paris, France; 2 Centre de Recherche des Cordeliers, Paris, France; 3 Université Pierre et Marie Curie-Paris6, UMR S 872, Paris, France; 4 The Global Fund to Fight AIDS, Tuberculosis and Malaria, WHO, Vernier – Geneva, Switzerland; The Fox Chase Cancer Center, United States of America

The long-standing quest for the development of vaccines that confer protection against highly mutable viruses such as HIV, hepatitis C, and influenza has elicited numerous structural and functional studies on virus-neutralizing human antibodies. These studies have aimed at translating the knowledge acquired on broadly neutralizing antibodies to the design of better immunogens for the induction of specific and protective immune responses. The last few months were marked by several seminal articles that investigate HIV-neutralizing human antibodies. These studies have characterized essential mechanistic details of the neutralization of HIV and imply that both exquisite specificity and degeneracy of the specificity of antibodies may be equally important for HIV neutralization. In this Opinion, we highlight and further discuss the potential of polyreactive (promiscuous) antibodies in defense against promptly evolving viruses. Despite having been somewhat neglected by mainstream immunologists in the last 20 years, polyreactive antibodies may come to light as new weapons against HIV.

## Strategies of HIV for Evading the Immune Response

HIV infection is characterized by the production of large amount of diverse virus-specific antibodies; these antibodies are, however, not capable of efficiently controlling virus propagation [1]. This is explained by the sophisticated immune evasion strategies of HIV [1], [2]. Members of the Retroviridae family possess an error-prone reverse transcriptase that introduces mutations at high frequency during reverse transcription of viral RNA into DNA [2]. Random mutations also affect the viral spike protein gp120, which mediates the attachment of HIV to the CD4 molecule on the host cells [2]. Indeed, the extraordinary diversity in the sequence of the surface motifs in gp120 explains the escape of HIV from effective neutralization by antibodies. The mutation-driven viral evolution is so intense that, in individual patients, versatile gp120 variants and even quasi-species of HIV are generated [1]. Paradoxically, the pressure exerted by the humoral immune response shapes gp120 diversity during the course of the infection. Another mechanism for immune evasion by HIV, defined as entropic masking [3], is related to the enormous structural flexibility of unbound gp120 [2]. Thus, gp120 displays many functionally irrelevant structural variants, a heterogeneity that misleads the immune system and skews the humoral immune response [3], [4]. Lastly, HIV also takes advantage of the immune inertness of host-derived glycans to shield binding epitopes on gp120 that are important for the virus, thus physically preventing antibody access [3], [5].

## HIV-Neutralizing Antibodies

Despite the ability of HIV to escape immune recognition, some individuals with long-standing HIV infection do generate broadly neutralizing antibodies; these antibodies were found to potently neutralize different HIV genetic variants [1], [6], [7]. Scientists were encouraged to characterize such immunoglobulins as templates to design novel vaccines. By using selection technologies, a number of broadly neutralizing human antibodies have been isolated [8]. The characterization of their interaction with gp120 or gp41 at the atomic level has allowed for the mapping of the sites on the viral surface that are sensitive to neutralization. Thus, regions on gp120 such as the CD4-binding site, the CD4-induced site (i.e., the site on gp120 which is exposed upon binding of CD4 to gp120), the co-receptor binding site, and the membrane-proximal external region (MPER) site on gp41 have been identified as essential targets for neutralizing antibodies [8]. Collectively, these endeavors have led to the emergence of the field of “structure-assisted rational vaccine design”, where structural information is used for the development of immunogens that elicit immune responses targeted specifically to sites on spike proteins that are vulnerable to neutralization by antibodies [9]. Recently published work [10], [11] describes such innovative strategies for the selection, from the peripheral blood of infected patients, of potent neutralizing antibodies to gp120 with broad clade specificity. The structural analyses of one of these antibodies showed that it binds to the CD4-binding site on gp120 [11]. The binding of this antibody mimics advantageously the interaction of CD4 with gp120, demonstrating how extreme optimization of antibody specificity by affinity maturation and accumulation of somatic mutations may result in high HIV neutralization potency. Moreover, the work of Zhou implies that, in order to be efficient, the immune response against HIV gp120 has to focus on a particular invariant site on the gp120 molecule but not be directed to neighboring epitopes.

Structural studies have shown that most of the antibodies that are able to neutralize HIV harbor atypical properties. Thus, the broadly neutralizing antibody 2G12 is able to swap its heavy chains in order to form an extended binding surface consisting of three binding sites, an efficient strategy for binding to carbohydrate moieties [12]. Other neutralizing antibodies were shown to possess unusually long and protruding heavy chain CDR3 [13], sulfated tyrosines [14], secondary structural motives in the CDRs [15], additional disulfide bridges, and/or N-linked glycosylation in the variable domains [11]. The most common feature of HIV-neutralizing antibodies, however, is the large number of somatic mutations in their variable domains [11]. The presence of these numerous mutations and the use of atypical protein modifications for efficient HIV neutralization suggests that the immune system is pushed to the limits of the diversity that it may generate by the mere introduction of variations in the polypeptide sequence, and that it explores alternative strategies to optimize antigenic neutralization. Interestingly, and notwithstanding the recent characterization of “extremely” broadly neutralizing antibodies, none of the described antibodies was shown to be able to neutralize all genetic variants of HIV [1], [10]. We propose that this is due to the exquisite specificity of these antibodies: minor variations in the target antigenic determinant of very specific antibodies, which are inherent to the elevated mutability of HIV, will be hardly accommodated by the antibodies. In other words, the tremendous energy the immune system spends in producing exquisitely specific and efficient neutralizing antibodies to HIV occurs at the cost of its capacity to adapt to the subsequent virus variants to be generated in the course of infection.

## Neutralization of HIV by Polyreactive Antibodies

In contrast to the report by Zhou et al. [11], which indicates that absolute epitope specificity is a necessity for the efficient neutralization of HIV, the study by Mouquet and colleagues [16] highlights the important role of polyreactive antibodies in controlling HIV infection. Polyreactivity is defined as the ability of an antibody molecule to bind several structurally unrelated antigens [17], [18]. In healthy individuals, at least 20% of circulating immunoglobulins are polyreactive. Polyreactive antibodies have been proposed as a first line of defense against pathogens [19]. Indeed, natural polyreactive antibodies have been demonstrated to synergize with the complement system in the opsonization of viruses and bacteria, thus directing the pathogens to secondary lymphoid organs and facilitating initiation of adaptive immune responses [20], [21]. Mouquet et al. [16] demonstrate that most of the anti-gp120 antibodies isolated from patients with high HIV-neutralizing titers are polyreactive. Interestingly, polyreactive antibodies in patients with HIV are also highly mutated, as opposed to most polyreactive antibodies in healthy individuals, which are in a germline configuration, thus suggesting a positive selection of B cell clones producing polyreactive antibodies with specificity for gp120. The authors propose that polyreactivity is utilized as a mechanism to increase the functional affinity (avidity) of the antibodies for the viral spikes. Thus, the simultaneous engagement (heteroligation) of gp140 (by one arm of an IgG) and of another yet unidentified structure on the viral membrane (by the other arm of the IgG) results in a great improvement in binding avidity [16]. Thus, this study confirmed the significance of antibody avidity in HIV neutralization that had been predicted earlier by Klein and Bjorkman [22]. This type of binding is especially advantageous in the case of HIV, as viral spikes are sparsely distributed on the viral membrane, and hardly neutralized by classical homo-ligation with monoreactive antibodies [22].

Previous studies have suggested that polyreactivity might improve the neutralization capacity of HIV-binding antibodies. Thus, antibodies 2F5 and 4E10, which are specific for MPER on gp41, were demonstrated to be polyreactive and also to recognize other proteins, i.e., histones, centromere B, Ro, and phospholipids [23]. These antibodies were shown to neutralize HIV by the simultaneous engagement of the membrane and gp41 [24]. Interestingly, the sole interaction with gp41 was not efficient for viral neutralization. Another polyreactive antibody with HIV-neutralizing properties is antibody 21c, which binds to the CD4-induced site on gp120 [25]. Efficient neutralization of the virus by 21c was, however, only possible following the simultaneous engagement of gp120 and CD4 [25].

The efficient neutralization of HIV by polyreactive antibodies may appear unexpected, given the fact that polyreactive antibodies are often considered to possess lower binding affinity than monoreactive antibodies [17], owing to the entropy penalty that arises from increased molecular flexibility of the polyreactive paratopes [26], [27]. In many cases of antibody–antigen interactions, however, unfavorable changes in entropy have been shown to be compensated by favorable changes in enthalpy of binding; the overall binding affinity is thus generally not considerably affected [28]–[32]. Importantly, affinity alone does not dictate the specificity and the function of antibodies, which is also largely determined by the biological context of the interaction [33].

In addition, a polyclonal response compensates for the possible vulnerability of individual polyreactive antibodies to statistical restrictions of their capacity to bind to highly flexible gp120.

## The Molecular Adaptability of Polyreactive Antibodies Can Contribute to HIV Neutralization

Taken together, the aforementioned studies on polyreactive HIV-neutralizing antibodies demonstrate the advantage of polyreactivity mostly as a way to gain in antigen-binding avidity. We further hypothesize that, in addition to a beneficial gain in avidity, polyreactive antibodies may better tolerate the elevated mutability of HIV. Many structural and biophysical studies have revealed that polyreactive antibodies, in contrast to highly specific antibodies, possess flexible and highly adaptable antigen-binding sites [26], [34]–[37]. Such high molecular dynamics of the antigen-binding sites of polyreactive antibodies creates an extended ensemble of conformations, with the capacity to adapt to structurally different epitopes. Such antibodies would thus accommodate much more easily structural and/or sequence alterations in promptly mutating proteins. In contrast, highly specific antibodies possess rigid and pre-optimized binding sites to interact with high specificity to given epitopes [37], [38]; small variations in the epitopes would be sufficient to abrogate the interaction of even very broadly and potently neutralizing antibodies. Indeed, biophysical and structural analysis of the interaction of antibodies with hen egg lysozyme, used as a model antigen, have revealed that polyreactive (less specific) antibodies are more tolerant to variations in their epitopes than highly specific antibodies [39]–[41].

Most of the surface of gp120 that is accessible to antibodies is covered with glycans. Hence, polyreactive antibodies that bind carbohydrates may also be of interest for virus neutralization. However, 2G12, a broadly neutralizing oligomannose-specific antibody proved to be less tolerant to variations in the glycosylation pattern of gp120 than the lectin concanavalin A [42]. Interestingly, plant lectins demonstrate intrinsic carbohydrate-binding polyreactivity, resulting in a better adaptation to different gp120 glycoforms [42], [43]. This suggests that lectin-like polyreactive antibodies may be a strategy towards targeting the diverse glycoforms of gp120 [43].

While all current HIV vaccination strategies, based on a structure-assisted rational vaccine design, aim at eliciting highly specific antibodies against sites that are vulnerable to neutralization, we suggest that perennial approaches to combat promiscuous mutable viruses such as HIV should also exploit the potential of promiscuous adaptive antibodies. To this end, vaccine strategies that elicit both highly specific antibodies and promiscuous HIV-specific antibodies, while limiting IgG-dependent transmission of HIV from dendritic cells to T cells in trans [44], should be explored.
